# Spontaneous high clonal expansion of Wilms’ tumor gene 1-specific cytotoxic T-lymphocytes in patients with Wilms’ tumor gene 1-expressing solid tumor

**DOI:** 10.1007/s00262-024-03862-8

**Published:** 2024-11-07

**Authors:** Soyoko Morimoto, Yukie Tanaka, Jun Nakata, Fumihiro Fujiki, Kana Hasegawa, Hiroko Nakajima, Sumiyuki Nishida, Akihiro Tsuboi, Naoki Hosen, Naoki Kagawa, Motohiko Maruno, Akira Myoui, Takayuki Enomoto, Shuichi Izumoto, Mitsugu Sekimoto, Naoya Hashimoto, Toshiki Yoshimine, Atsushi Kumanogoh, Yusuke Oji, Yoshihiro Oka, Haruo Sugiyama

**Affiliations:** 1 Department of Cancer Stem Cell Biology, Osaka University Graduate School of Medicine, Osaka, Japan; 2 Department of Cancer Immunotherapy, Osaka University Graduate School of Medicine, Osaka, Japan; 3 Department of Molecular Microbiology and Immunology, Graduate School of Medical and Dental Sciences, Institute of Science Tokyo, Tokyo, Japan; 4 Department of Clinical Laboratory and Biomedical Sciences, Osaka University Graduate School of Medicine, Osaka, Japan; 5 Laboratory of Cellular Immunotherapy, World Premier International Research Center Initiative Immunology Frontier Research Center (WPI-IFReC), Osaka University, Osaka, Japan; 6 Tumor Immunotherapy Program, Princess Margaret Cancer Centre, University Health Network, Toronto, ON Canada; 7 Department of Cancer Immunology, Osaka University Graduate School of Medicine, Osaka, Japan; 8 Strategic Global Partnership & X (Cross)-Innovation Initiative Graduate School of Medicine, Osaka University and Osaka University Hospital, Osaka, Japan; 9 Department of Respiratory Medicine and Clinical Immunology, Osaka University Graduate School of Medicine, Osaka, Japan; 10 Center for Advanced Modalities and Drug Delivery System, Osaka University, Osaka, Japan; 11 Department of Hematology and Oncology, Osaka University Graduate School of Medicine, Osaka, Japan; 12 Integrated Frontier Research for Medical Science Division, Institute for Open and Transdisciplinary Research Initiatives, Osaka University, Osaka, Japan; 13 Department of Neurosurgery, Osaka University Graduate School of Medicine, Osaka, Japan; 14 Department of Neurosurgery, Yukioka Hospital, Osaka, Japan; 15 Medical Center for Translational Research, Department of Medical Innovation, Osaka University Hospital, Osaka, Japan; 16 Department of Obstetrics and Gynecology, Osaka University Graduate School of Medicine, Osaka, Japan; 17 Center for Genetic Medicine, Itami City Hospital, Hyogo, Japan; 18 Department of Neurosurgery, Hyogo College of Medicine, Hyogo, Japan; 19 Department of Neurosurgery, Kindai University Nara Hospital, Nara, Japan; 20 Department of Gastroenterological Surgery Graduate School of Medicine, Osaka University, Osaka, Japan; 21 Department of Surgery, Minoh City Hospital, Osaka, Japan; 22 Department of Neurosurgery, Graduate School of Medical Science, Kyoto Prefectural University of Medicine, Kyoto, Japan; 23 Endowed Research Department of Clinical Neuroengineering, Global Center for Medical Engineering and Informatics, Osaka University, Osaka, Japan; 24 Iseikai Medical Corporation, Osaka, Japan

**Keywords:** WT1, WT1_126_-CTLs, Single-cell, TCR repertoire, Clonality

## Abstract

Wilms’ tumor protein 1 (WT1)-targeted immunotherapy has been used in patients with leukemia and solid tumors. However, the spontaneous WT1-specific immune response before WT1 peptide vaccination in patients with WT1-expressing tumors (PTs) remains unclear. Therefore, we investigated whether WT1-specific cytotoxic CD8^+^ T-lymphocytes (CTLs) are clonally expanded in the peripheral blood outside of tumor sites. Clonal expansion of WT1_126_ peptide (a.a.126–134)-specific CTLs (WT1_126_-CTLs) was compared between seven PTs and five healthy volunteers (HVs), and their T-cell receptors (TCRs) were analyzed at the single-cell level. Overall, 433 and 351 TCR β-chains of WT1_126_-CTLs were detected from PTs and HVs, respectively, and complementarity-determining region 3 was sequenced for clonality analysis. The frequencies of WT1_126_-CTLs were higher in human leukocyte antigen (HLA)-A*02:01^+^ PTs than in HLA-A*02:01^+^ HVs, although the difference was not statistically significant. WT1_126_-CTLs of differentiated types, including memory and effector, were higher in PTs than in HVs; whereas, those of the naïve type were higher in HVs than in PTs. WT1_126_-CTL clonality was significantly higher in PTs than in HVs. Furthermore, the frequency of effector WT1_126_-CTLs positively correlated with WT1_126_-CTL clonality in PTs; whereas, the frequency of naïve phenotype WT1_126_-CTLs tended to be negatively correlated with clonality. In conclusion, these results suggest that the WT1 protein in tumor cells is highly immunogenic, thereby stimulating endogenous naïve-type WT1_126_-CTLs and enabling them to clonally expand and differentiate into effector-type WT1_126_-CTLs.

## Introduction

Tumor-associated antigen (TAA)-specific cytotoxic T-lymphocytes (CTLs) are the main effectors of immunological attack on tumor cells. To date, several investigations have been performed to analyze tumor-infiltrating lymphocytes in patients with solid tumors. Some of these studies have shown T-cell receptor (TCR) sequence-based clonal expansion of TAA-specific CTLs at tumor sites, indicating that TAA-specific CTLs, which are activated and expanded, accumulate at tumor sites. Comparative evaluation of spontaneous clonal proliferation of TAA-specific CTLs in peripheral blood (PB), a non-tumor site in patients with various types of solid tumors, and clonal proliferation of TAA-specific CTLs in healthy human PB will hopefully provide us with important insights into understanding anti-tumor immunity.

Wilms’ tumor gene 1 (*WT1*) is expressed in various types of solid tumors [1, 2] and hematological malignancies [3] and plays important roles in oncogenesis [4]. In carcinogenesis, WT1 is considered to have a potential oncogenic role by promoting cell proliferation [5–7] and motility [8], while inhibiting apoptosis [9] through its overexpression. Consequently, WT1 has been identified as one of a potential target antigen for cancer immunotherapy, and had previously been selected as the most promising one of the 75 TAAs [10]. We previously identified WT1-CTL epitopes and WT1_126_ and modified WT1_235_ (a.a. 235–243) peptides that can induce CTLs with killing activity against WT1-expressing tumors by restricting human leukocyte antigen (HLA)-A*02:01 and HLA-A*24:02, respectively [11, 12]. These HLA class I types are frequently found in humans, and both our research and that of others have reported a series of successful clinical studies using these WT1 peptide-based vaccine therapies for patients with solid tumors [13–15] and hematological malignancies [15–17].

Compared to the lack of reports showing clonality of TAA-specific CTLs in the PB of patients with solid tumors, as mentioned above, several investigations, including ours, have reported clonality of WT1-specific CTLs in the PB or bone marrow (BM) of patients with acute myeloid leukemia (AML), in which PB and BM are the areas where abundant leukemic cells exist, that is, tumor sites [18–20]. Therefore, this study aimed to investigate whether WT1-specific CTLs are clonally expanded in the PB outside the tumor site of patients to comprehensively understand the nature of the anti-cancer immune response in patients with solid cancer. If the clonal expansion of WT1-specific CTLs in the PB of patients is demonstrated, the clonal expansion of CTLs may also exist in tumor-draining lymph nodes (LNs) that are non-tumor sites, such as the PB. The expectation of WT1 peptide vaccine therapy is to artificially induce and activate WT1-specific CTLs through the migration of dendritic cells carrying intradermally administered WT1 peptides from the skin to LNs, and antigen presentation to WT1-specific CTLs in LNs. If WT1-specific CTLs with advanced differentiation are already present in the PB of patients with solid tumors prior to treatment and are proliferating clonally, administration of the WT1 peptide vaccine is expected to rapidly activate these CTLs and further promote clonal proliferation. The existence of long-term viable WT1-specific CD8^+^ T cells is important for the long-term anti-tumor effect. However, the pre-existence of WT1-specific effector CD8^+^ T cells that can rapidly attack tumors after the start of WT1 peptide vaccine therapy is an important factor for the success of this therapy. For this reason, we believe that proof of clonal expansion prior to WT1 peptide vaccination in the PB of patients with solid tumors will strengthen the expectation of WT1 peptide vaccine therapy in these patients.

## Materials and methods

### Samples

Peripheral blood mononuclear cells (PBMCs) were isolated from heparinized blood samples obtained from seven HLA-A*02:01 patients with solid tumors and five HLA-A*02:01 healthy volunteers (HVs) prior to the WT1-235 peptide vaccination by using Ficoll–Hypaque gradient centrifugation in Lymphocyte Separation Solution (Nacalai Tesque, Inc., Kyoto, Japan) and cryopreserved in liquid or gas-phase nitrogen until use. Tumor cells of patients were analyzed for WT1 protein expression using immunohistochemical analysis, as previously described [21]. Patients were enrolled in the University Hospital Medical Information Network (UMIN) Clinical Trials (UMIN number: UMIN000002001) on May 24, 2009. This observational study was carried out by using the patients' samples approved by the Institutional Review Board for Clinical Research of Osaka University Hospital on June 15, 2012 (IRB number: 11293). Written informed consent was obtained from all patients and HVs. All mandatory laboratory health and safety procedures were complied with during the study. Tumor response was defined based on the investigator's assessment according to the Response Evaluation Criteria for Solid Tumors.

### WT1-tetramer, antibodies, and flow cytometry

The PE-labeled HLA-A*02:01 WT1_126-134_ (RMFPNAPYL) tetramer (WT1_126_ tetramer) was purchased from MBL Co., Ltd. (Nagoya, Japan). WT1_126_-specific CTLs were detected using the WT1_126_ tetramer and other monoclonal antibodies (mAbs) as previously described [22]. Briefly, thawed PBMCs were rested for 1.5 h and stained with WT1_126_ tetramer at 37 °C for 30 min. Subsequently, these PBMCs were stained with anti-human mAbs at 4 °C for 25 min. The following mAbs were used: anti-CD4-FITC, anti-CD16-FITC, and anti-CD45RA-APC (BioLegend, San Diego, CA, USA); anti-CD19-FITC and anti-CCR7-PE-Cy7 (BD Pharmingen, San Diego, CA, USA); anti-CD3-PerCP, anti-CD8-APC-Cy7, and anti-CD14-FITC (BD Biosciences, San Jose, CA, USA); and anti-CD56-FITC (eBioscience, San Diego, CA, USA). After staining, WT1_126_-specific CTLs were directly sorted into polymerase chain reaction tubes containing a cDNA reaction-mix solution using a FACSAria (BD Biosciences). WT1_126_-specific CTLs were stained with anti-PD-1-PE-Cy7 (EH12.2H7; BioLegend, San Diego, CA, USA), anti-LAG-3-APC (3DS223H; San Diego, CA, USA), and anti-Tim-3-APC (F38-2E2; San Diego, CA, USA) antibodies to detect the expression of exhaustion markers in WT1_126_-specific CTLs. Anti-mouse IgG1, κ-PE-Cy7 (BioLegend), and -APC (TONBO Biosciences) (MOPC-21; San Diego, CA, USA) were used as isotype controls. Data were analyzed using FlowJo 7.6.5 and 10.9.0 software (FlowJo LLC, Ashland, OR, USA).

### Single-cell sorted TCR repertoire analysis

cDNA was synthesized from single-cell sorted WT1-specific CTLs, as previously described [22]. Complementarity-determining region 3 (CDR3) amino acid sequences of TCR β-chains in individually sorted single-cell CTLs were analyzed using IMGT/V-QUEST (https://www.imgt.org/IMGT_vquest/input).

### TCR repertoire clonality

Clonality (*C*) was normalized by entropy (*E*), which was calculated using Shannon’s definition, as follows [23]:

$$E = - \sum\limits_{i = 1}^{N} {fi\log_{2} } fi$$

$$C = 1 - \frac{E}{{\log_{2} (N)}}$$

where *fi* represents the ratio occupied by each clone in all analyzed cells, and N represents the total number of clones.

## Statistical analysis

The Mann–Whitney U test was used to evaluate the differences in the frequency of WT1_126_-specific CTLs and clonality between patients with WT1-expressing tumors (PTs) and HVs. A two-way ANOVA followed by Sidak’s multiple comparison test was used to evaluate the phenotypic differences in WT1_126_-specific CTLs between PTs and HVs. Pearson’s correlation was used to calculate the significance of the correlations between the phenotypes and clonality of WT1_126_-specific CTLs. All statistical analyses were performed using GraphPad Prism versions 7 and 10 (GraphPad Software Inc., La Jolla, CA, USA). *P*- values < 0.05 were considered significant in all analyses.

## Results

### Frequencies of WT1_126_-specific CTLs in PB

**Fig. 1:**
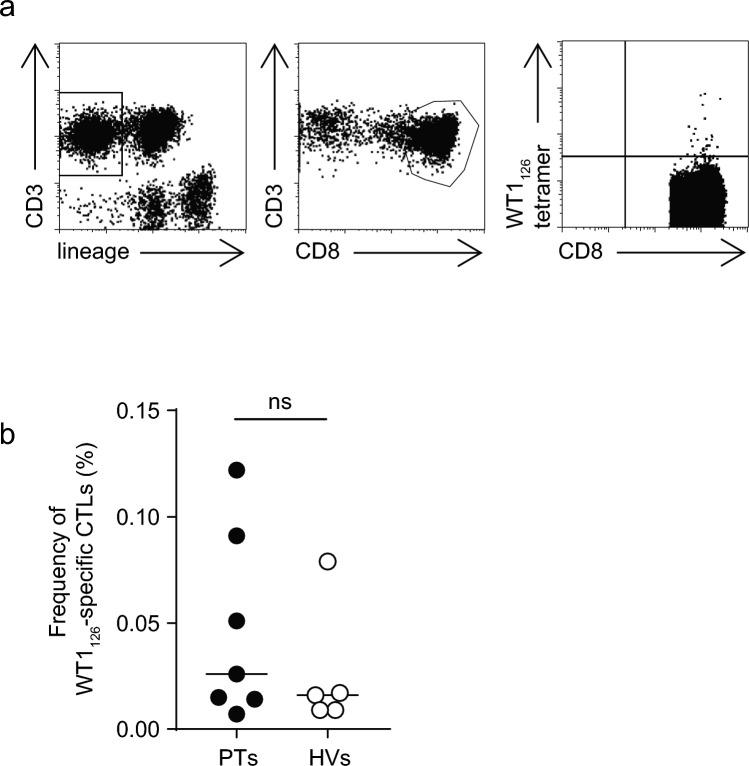
Frequency of WT1_126_-specific CTLs in CD8^+^ T cells. **a** WT1_126_-specific CTLs were defined by flow cytometry as CD3^+^, CD8^+^, WT1_126_ tetramer^+^, and lineage markers (CD4, CD14, CD16, CD19, and CD56)-negative cells. **b** Frequencies of WT1_126_-specific CTLs in CD8^+^ T cells. Bars indicate the median values of the frequencies. No significant difference was found in the frequencies. WT1; Wilms’ tumor protein 1; CTLs, cytotoxic T-lymphocytes; PTs, patients with WT1-expressing tumor; HVs, healthy volunteers; ns, not significant

The characteristics of seven cancer PTs and five HVs are shown in Table 1 [22]. The median age of PTs and HVs were 53 years (range 18–73 years) and 25 years (range 23–45 years), respectively. All PTs underwent surgery, and the timing of PBMCs collection is indicated by the period following prior treatment. We defined WT1_126_-specific CTLs as CD3^+^, CD8^+^, WT1_126_ tetramer^+^, and lineage marker (CD4, CD14, CD16, CD19, and CD56)-negative cells (abbreviated as WT1_126_-CTLs) (Fig. 1a). Because the frequencies of WT1_126_-CTLs in PB are generally as low as 1/10,000 to 1/1,000 of those in CD8^+^ T cells, we performed fluorescence-activated cell-sorting in at least one million PBMCs to accurately measure the frequencies and obtain WT1_126_-CTLs sufficient to analyze their TCR β-chain variable repertoires. The frequencies of WT1_126_-CTLs were 0.007–0.122% (median: 0.026%) and 0.009–0.079% (median: 0.016%) in PTs and HVs, respectively, without statistically significant difference (Fig. 1b) [22].

**Table 1:** Characteristics of the patients and healthy volunteers

No	Age, y/sex	Disease	Prior treatment	Period after prior treatment (weeks)	WT1_126_-specific CTL (%)	Phenotype of WT1_126_-specific CTL (%)				WT1_126_-specific CTLs' count of repertoire analysis	Clinical response to WT1235m peptide vaccination
						N	CM	EM	E		
PT−1	33/M	GBM	Ope/RT	5	0.053	11.6	2.3	46.5	39.5	59	PR
PT−2	56/F	GBM	Ope/RT	6	0.015	25.7	22.9	40.0	11.4	66	PD*
PT−3	28/M	GBM	Ope/RT/Chemo	16	0.007	30.3	6.1	42.4	21.2	46	SD
PT−4	18/M	PNET	Ope/RT/Chemo/auto-PBSCT	13	0.122	2.8	2.8	50.0	44.4	66	PD
PT−5	53/F	Ovarian cancer	Ope/Chemo	18	0.025	41.5	3.7	36.6	18.3	88	SD
PT−6	73/F	Cecal cancer	Ope/Chemo	4	0.091	19.2	16.4	37.0	27.4	73	PD
PT−7	73/M	Thyroid cancer	Ope	7	0.015	7.1	0.0	7.1	85.7	35	SD
HV−1	23/F	–	–	–	0.009	53.3	13.3	13.3	20.0	53	–
HV−2	45/M	–	–	–	0.016	36.7	3.3	26.7	33.3	57	–
HV−3	24/F	–	–	–	0.009	63.4	19.5	14.6	2.4	77	–
HV−4	25/F	–	–	–	0.017	58.3	8.3	33.3	0.0	79	–
HV−5	37/M	–	–	–	0.079	58.1	7.7	25.6	8.6	85	–

*GBM* glioblastoma multiforme, *PNET* primitive neuroectodermal tumor, Ope, operation, *RT* radiation therapy, Chemo, chemotherapy, *auto-PBSCT* autologous peripheral blood stem cell transplantation, *N* Naïve, *CM* Central Mmemory, *EM* Effector Memory, *E* Effector, *PR* partial response, *SD* stable disease, *PD* progression disease, PD*, long-lasting PD

### Differences in the phenotypes of WT1_126_-specific CTLs between PTs and HVs

**Fig. 2:**
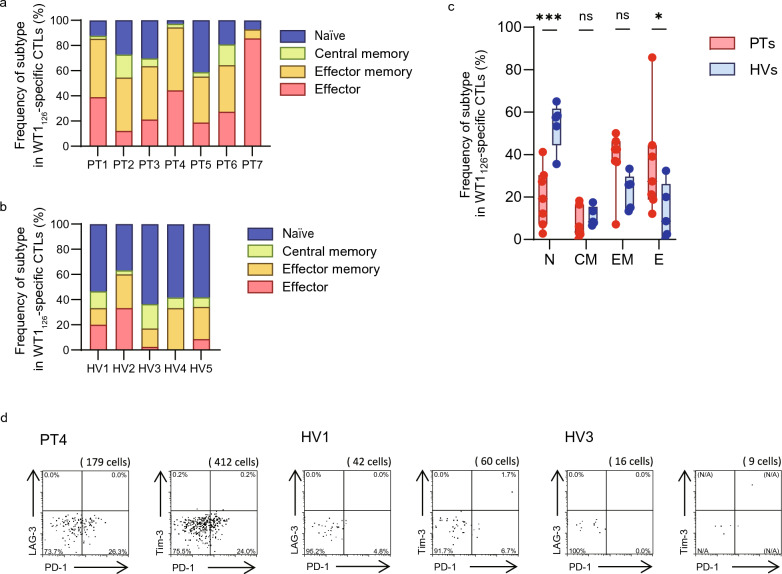
Phenotypes of WT1_126_-specific CTLs Phenotypes of WT1_126_-specific CTLs in seven PTs (**a**) and five HVs (**b**). WT1_126_-specific CTLs were classified into four distinct subtypes based on the four differentiation stages according to the cell surface expression of CD45RA and CCR7 as follows: (i) naïve cells, CD45RA^+^ CCR7^+^; (ii) central memory, CD45RA^−^ CCR7^+^; (iii) effector memory, CD45RA^−^ CCR7^−^; and (iv) effector, CD45RA^+^ CCR7^−^. **c** Subtype frequency in WT1_126_-specific CTLs. Box plots represent median ± 25th percentile, with whiskers representing min/max values. Red boxplots: PTs; blue boxplots: HVs. *P*-values were obtained using a two-way ANOVA followed by Sidak’s multiple comparison test. **d** The exhaustion state of WT1_126_-specific CTLs in PT4, HV1, and HV3. *P < 0.05, ***P < 0.001. ns, not significant. WT1, Wilms’ tumor protein 1; CTLs, cytotoxic T-lymphocytes; PTs, patients with WT1-expressing tumor; HVs, healthy volunteers; N/A, not assessable (less than 10 cells)

We examined the phenotypes of the WT1_126_-specific CTLs [22]. WT1_126_-specific CTLs were categorized into four distinct subtypes, corresponding to the four differentiation stages, based on the cell surface expression of CD45RA and CCR7: (i) naïve cells, CD45RA^+^ CCR7^+^; (ii) central memory, CD45RA^−^ CCR7^+^; (iii) effector memory, CD45RA^−^ CCR7^−^; and (iv) effector, CD45RA^+^ CCR7^−^. Most of the WT1_126_-specific CTLs in all seven PTs exhibited higher percentages of effector memory (36.6–50.0%, median: 40.0%) and effector (11.4–85.7%, median: 27.4%) phenotypes (Fig. 2a). Notably, WT1_126_-specific CTLs of 86.0%, 94.4%, and 92.8% in PTs 1, 4, and 7, respectively, showed extremely differentiated phenotypes (effector memory and effector). However, most of the WT1_126_-specific CTLs in all five HVs predominantly exhibited a naïve phenotype (36.7–63.4%, median: 58.1%) (Fig. 2b). Significant differences were found in the proportions of CTLs with naïve (P = 0.0008) and effector phenotypes (P = 0.0336) between PTs and HVs (Fig. 2c). Cell surface expression of exhausted markers in WT1_126_-specific CTLs was assessed using specimens for which abundant numbers of samples had been stored. The frequencies of exhausted PD-1^+^ LAG-3^+^ and PD-1^+^ Tim-3^+^ T cells in PT4 were 0.0% and 0.2%, respectively, which were comparable to those in HVs (Fig. 2d). Conversely, the frequency of PD-1 single positive in PT4 was higher than that of HV1 and HV3.

### Oligoclonal expansion of WT1_126_-CTLs

**Fig. 3:**
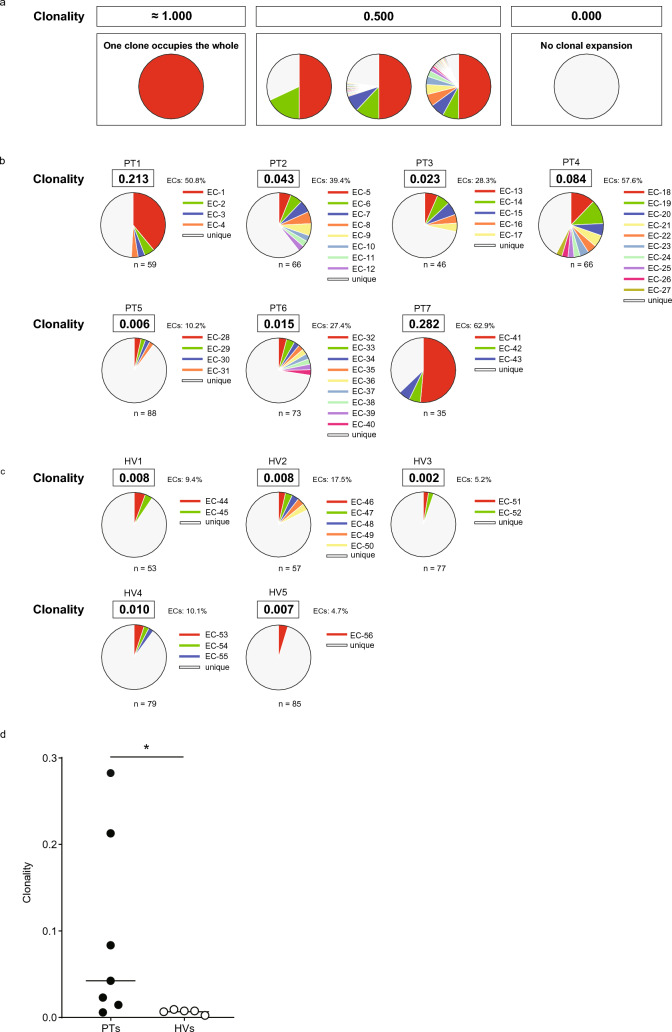
T-cell repertoire clonality of WT1_126_-specific CTLs (a) Schema of the concept of TCR repertoire clonality. In cases where only one WT1-CTL clone occupies the whole, the clonality is calculated to be infinitely close to 1.000 (0.9999) but not 1.0. A clonality of 0.000 indicates no clonal expansion. Clonality 0.500 includes various types of clonal expansion. The clonalities of WT1_126_-specific CTLs are shown in PTs (b) and HVs (c). Individual clones are shown in different colors. Sequences with clonally expanded clones in each donor are shown in the same color except light gray. Light gray indicates CTLs with unique amino acid residues, that is, unexpanded CTL clones. (d) Graphical representation of the clonality of WT1_126_-specific CTLs in PTs (n = 7) and HVs (n = 5). Bars indicate the median value of the clonality. Differences in clonality between PTs and HVs were significant (*P < 0.05). *P*-values were obtained using the Mann–Whitney U test. WT1; Wilms’ tumor protein 1; CTLs, cytotoxic T-lymphocytes. PTs, patients with WT1-expressing tumor; HVs, healthy volunteers; TCR, T-cell receptors

We examined the TCR repertoire of WT1_126_-CTLs by sequencing the CDR3 regions of TCR β-chains of the individual single-cell sorted CTLs to investigate the difference in the diversity of WT1_126_-CTLs between PTs and HVs. A total of 59, 66, 46, 66, 88, 73, and 35 CDR3 sequences were obtained from PTs 1, 2, 3, 4, 5, 6 and 7, respectively, and 53, 57, 77, 79, and 85 CDR3 sequences from HVs 1, 2, 3, 4 and 5, respectively (Table 1). The CDR3 usage frequency was considered to be accurately determined since the amplification efficiency of the CDR3 sequences was > 80%. Figure 3a schematically shows the concept of TCR repertoire clonality (abbreviated as clonality). In the case where the WT1-CTLs were occupied by only one clone, the clonality was calculated to be infinitely close to 1.000 (0.9999) but not 1.0; whereas, clonality 0 meant that no clones were present. Figures 3b and 3c show the usage frequencies of CDR3 sequences of TCR β-chains of the WT1_126_-CTLs and the clonality in PTs and in HVs, respectively. TCR β-chains detected more than twice in each sample were considered expanded clones (ECs). PTs not only had more types of ECs than HVs, but in some cases, such as PT1 and PT7, a single EC accounted for a high percentage. Figure 3d graphically shows the clonality of PTs and HVs. Clonality was significantly higher in PTs than in HVs (P < 0.05).

### Clear correlation between effector phenotype and clonality of WT1_126_-specific CTLs in PTs

**Fig. 4:**
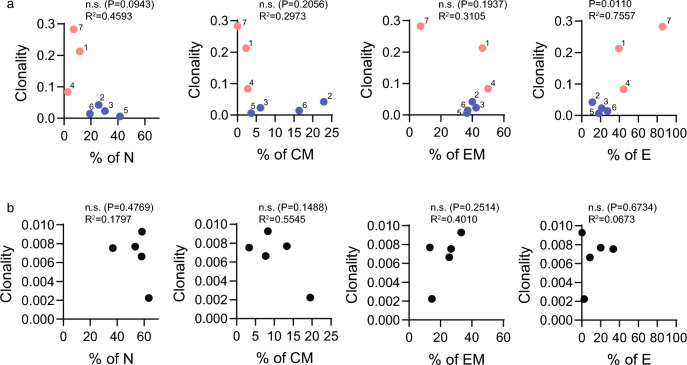
Correlation between the subtype of WT1_126_-specific CTLs and clonality Correlation between the subtype of WT1_126_-specific CTLs and clonality is shown for PTs (n = 7) (**a**) and HVs (n = 5) (**b**). Small numbers represent patient numbers. Red circles represent the top three PTs in terms of clonality, and blue circles represent the remaining PTs. R^2^ denotes Pearson’s correlation. P values were obtained using two-sided t tests. WT1; Wilms’ tumor protein 1; CTLs, cytotoxic T-lymphocytes. PTs, patients with WT1-expressing tumor; HVs, healthy volunteers; N, naïve (CD45RA^+^ CCR7^+^); CM, central memory (CD45RA^−^ CCR7^+^); EM, effector memory (CD45RA^−^ CCR7^−^); E, effector (CD45RA^+^ CCR7.^−^)

We evaluated the correlation between the phenotype and clonality of WT1_126_-specific CTLs in PTs and HVs (Fig. 4). The frequency of the effector phenotype of WT1_126_-specific CTLs positively correlated with the clonality of WT1_126_-specific CTLs in PTs (P = 0.0110, R^2^ = 0.7557); whereas, the frequency of the naïve phenotype of WT1_126_-specific CTLs in PTs tended to be negatively correlated with the clonality of WT1_126_-specific CTLs (P = 0.0943, R^2^ = 0.4593) (Fig. 4a). However, the frequencies of the central memory and effector memory phenotypes did not correlate with clonality, and no correlation was found between the phenotype and clonality of WT1_126_-specific CTLs in HVs (Fig. 4b). Therefore, these results indicate that WT1_126_-specific CTLs proliferated in the immune response against the WT1 antigen of tumor cells in PTs, may differentiate from naïve to terminal effector phenotypes, and may clonally expand during this cell differentiation in association with the continuous proliferation of WT1_126_-specific CTLs, leading to higher clonality of WT1_126_-specific CTLs.

## Discussion

This study demonstrated for the first time that the clonality of WT1_126_-specific CTLs was significantly higher in PTs than in HVs, although the frequency of WT1_126_-specific CTLs did not differ significantly between the groups. Most of the WT1_126_-specific CTLs in all seven PTs exhibited effector memory and effector phenotypes; whereas, most of the WT1_126_-specific CTLs in all five HVs exhibited naïve phenotypes. The frequency of the effector phenotype of WT1_126_-specific CTLs in PTs positively correlated with CTL clonality, whereas that of the naïve phenotype of CTLs negatively correlated with clonality. No correlation was found between the phenotype and clonality of WT1_126_-specific CTLs in HVs.

In mouse models, the clonal expansion of T cells induced by strong TCR signals, including the foreign antigen OVA peptide, has been reported to exhibit differentiated phenotypes, such as effector memory and effectors [24, 25]. However, there have been no reports of spontaneous and clonal proliferation of CTLs specific for overexpressed protein antigens such as WT1 in the PB of solid tumor patients. All patients underwent surgery, chemotherapy, and/or radiotherapy before PBMC samples were collected. Therefore, a large amount of WT1 antigen was likely released from collapsed WT1-expressing tumor cells during these treatments, which endogenously induced WT1-specific CTLs, followed by clonal expansion in association with cell proliferation and differentiation from naïve to memory phenotypes. The early administration of the WT1 peptide vaccine after tumor collapse is expected to cause rapid clonal expansion of WT1-specific CTLs, which can attack WT1-expressing tumors and lead to a favorable clinical response. Moreover, PTs 1 and 7, who received the modified WT1_235_ peptide vaccine relatively early (5–7 weeks after pretreatment) and showed high clonality, achieved partial response and stable disease, respectively. This suggests epitope spreading due to tumor disruption from prior therapy.

Epitope and antigen spreading have been reported to be favorable prognostic factors for many cancer immunotherapies [26–28]. However, no clear predictors of favorable clinical response were found in our study because of the small number of patients analyzed. Therefore, we hope to increase the number of patients analyzed in the near future to identify prognostic factors. In all HVs, a small number of WT1_126_-specific CD8^+^ T cells showed clonality (Fig. 3c). This clonality may indicate the existence of a tumor immune surveillance system involving WT1-specific CD8^+^ T cells. Cancer immunoediting is an essential process where the immune system suppresses or promotes tumor development via the following three processes: elimination, equilibrium, and escape [29]. During the elimination process, the innate and adaptive immune systems cooperate to suppress tumors. Tumor-specific CD8^+^ T cells recognize and destroy tumor antigen-expressing tumor cells. This immunosurveillance system leads to tumor disappearance. In HVs, in whom clinically apparent tumors are absent, the tumor elimination process may effectively operate and thoroughly eradicate newly appearing tumors via tumor-specific CD8^+^ T cells.

Several studies have reported the clonal expansion of tumor-infiltrating lymphocytes specific to TAAs, such as Melan-A, MART-1, and NY-ESO-1, in patients with solid tumors [30–32] and WT1-specific CD8^+^ T cells in the PB and BM of patients with AML [19, 33]. However, no reports of clonal expansion of TAA-specific CD8^+^ T cells in the PB of patients with solid tumors before tumor antigen-targeting immunotherapy administration have been documented. CD8^+^ T cells recognize TAAs presented by antigen-presenting cells, such as dendritic cells in the LNs, and infiltrate the tumor site via the peripheral bloodstream. Since circulating CD8^+^ T cells serve as a source of tumor-infiltrating CD8^+^ T cells, exhaustion of circulating CD8^+^ T cells is a key factor in determining the cytotoxicity of tumor-infiltrating CD8^+^ T cells. Oliveira et al. demonstrated that the same clones as those in circulating CD8^+^ T cells could be detected in tumor-infiltrating CD8^+^ T cells and that the exhaustion state of the clones may be an indicator of the patient’s disease status and responsiveness to immune checkpoint blockade [34]. Therefore, we evaluated the exhaustion state of WT1_126_-specific CD8^+^ T cells from PT4 cells, which contained abundant samples for further analysis. The frequencies of exhausted PD-1^+^ LAG-3^+^ and PD-1^+^ Tim3^+^ T cells were 0.0% and 0.2%, respectively, which were comparable to those in healthy donors (Fig. 2d). These results indicate that the clonally expanded WT1_126_-specific CD8^+^ T cells in the PB of the patient were not exhausted and that they could be activated through subsequent WT1 peptide vaccination, leading to tumor reduction. Therefore, phenotypic analysis of clonally expanded WT1-specific CD8^+^ T cells may be useful for predicting the clinical effect of the WT1 peptide vaccine. However, due to the limited sample size in our study, no definitive answer exists, and further research is required to address this issue.

In conclusion, this is the first demonstration of spontaneous clonal expansion of WT1-specific CTLs in circulating T cells in patients with solid tumors. These results suggest that the WT1 protein in tumor cells is highly immunogenic, thereby stimulating endogenous naïve-type WT1_126_-CTLs and enabling them to clonally expand and differentiate into effector-type WT1_126_-CTLs. The presence of clonally expanded WT1_126_-specific CTLs with advanced differentiation stages in the PB of solid tumor patients prior to WT1 peptide vaccine therapy suggests that these CTLs may rapidly trigger tumor attack after WT1 peptide vaccine administration. Although our study cannot draw definitive conclusions due to the limited number of samples, further analysis of a larger number of samples may provide us with not only proof-of-concept for ongoing WT1 peptide vaccine clinical trials, but also a basis for predicting therapeutic efficacy before initiating WT1 peptide vaccine therapy.

## Data Availability

The datasets generated during and/or analyzed during the current study are available from the corresponding author on reasonable request. No datasets were generated or analyzed during the current study.

## Abbreviations

APC: Allophycocyanin
CTLs: Cytotoxic CD8^+^ T-lymphocytes
Cy7: Cyanine7
FITC: Fluorescein isothiocyanate
HLA: Human leukocyte antigen
HVs: Healthy volunteers
PTs: Patients with WT1-expressing tumors
PE: Phycoerythrin
PerCP: Peridinin-chlorophyll protein complex
TCR: T-cell receptor
WT1: Wilms’ tumor protein 1
